# Exploring the concerns of persons with disabilities in Western Zambia

**DOI:** 10.4102/ajod.v7i0.446

**Published:** 2018-11-29

**Authors:** Shaun Cleaver, Helene Polatajko, Virginia Bond, Lilian Magalhães, Stephanie Nixon

**Affiliations:** 1 School of Physical and Occupational Therapy, McGill University, Canada; 2 International Centre for Disability and Rehabilitation, University of Toronto, Canada; 3 Department of Occupational Science and Occupational Therapy, University of Toronto, Canada; 4 Rehabilitation Sciences Institute, University of Toronto, Canada; 5 Social Science Unit, ZAMBART, Zambia; 6 Department of Global Health and Development, London School of Hygiene and Tropical Medicine, United Kingdom; 7 Department of Occupational Therapy, Federal University of São Carlos, Brazil; 8 Department of Physical Therapy, University of Toronto, Canada; 9 Dalla Lana School of Public Health, University of Toronto, Canada

## Abstract

**Background:**

Understandings of disability are rooted in contexts. Despite the world’s significant contextual diversity, postcolonial power dynamics allow influential actors from the global North to imagine that most people across the global South understand disability in one generalised way. When it informs programmes and services for persons with disabilities in the global South, this imagining of a single generalised view could reduce effectiveness while further marginalising the people for whom the programmes and services were designed.

**Objectives:**

In the interest of better understanding a contextually grounded meaning of disability, we explored the expressed concerns of two organisations of persons with disabilities and their members in Western Zambia.

**Method:**

In this qualitative constructionist study, data collection focused upon life with a disability and services available to persons with disabilities. Data were collected through 39 individual interviews and eight focus group discussions with 81 members of organisations of persons with disabilities. Data were analysed thematically.

**Results:**

The participants’ main expressed concern was poverty. This concern was articulated in terms of a life of suffering and a need for material resources. Participants linked poverty to disability in two ways. Some participants identified how impairments limited resource acquisition, resulting in suffering. Others considered poverty to be an integral part of the experience of disability.

**Conclusion:**

This study contributes to literature on disability theory by providing a contextually grounded account of a particular understanding of disability and poverty. The study also contributes to disability practice and policymaking through the demonstration of poverty as the main concern of persons with disabilities in this context.

## Introduction

In the contemporary postcolonial world, the global South and the global North are connected through mechanisms patterned by the ongoing legacies of colonialism (Grech 2015). These mechanisms include political and economic structures that enable wealth and governance power to be drawn away from the majority populations in the low- and middle-income countries of Africa, Asia and Latin America, to instead concentrate in the global North. Indeed, it is the ongoing legacies of colonialism that make possible the phenomenon of the global South, in which diverse people and places share the common experience of having been colonised by imperial powers.^1^

With respect to disability, it is well accepted that the majority of persons with disabilities (PWDs) live in the global South (World Health Organisation & World Bank 2011). Populations of PWDs living in the global South are generally seen to face circumstances that are of a different nature than their counterparts with disabilities in the global North (Singal & Muthukrishna 2014). The often-difficult circumstances experienced by PWDs in the global South have attracted the interest of several influential ‘international’ actors including aid agencies, non-governmental organisations and researchers from academic fields such as international development, global health and disability studies.

Although the actors attracted to the circumstances of PWDs in the global South are international in the sense of being involved in multiple countries, they are often guided by the world views and priorities of the global North. One example of this would be a disability-focused non-governmental organisation that competes for grant money from national development agencies in the global North to fund programmes that operate in multiple countries of the global South. From the perspective of ‘international’ actors, it is possible to imagine the experiences of disability from diverse cultures and peoples according to a small number of homogenised narratives of disability in the global South (Miles 2007).

The dominant expression of a homogenised narrative of disability in the global South might be referred to as ‘the traditional model of disability’. When disability in the global South is viewed through the lens of this traditional model, a myriad of peoples and cultures are seen to understand disability as a supernatural phenomenon initiated by curses, compelling individuals to avoid experiencing shame by hiding or killing their family members with disabilities (Ingstad 1999). It is true that there are reports that substantiate some elements of this supposed traditional model (e.g. Bamu, De Schauwer & Van Hove 2016; Héraud 2005; Muderedzi & Ingstad 2011). Nonetheless, it is unjustifiable to use these observations from particular places to support a generalised view about how disability is understood in the global South (Ingstad 1999). Problematically, such a generalised view can allow influential actors to overlook the actual world views and priorities of PWDs in the global South. This generalised view can therefore be a distraction from important historical and contextual realities, further disempowering the supposed beneficiaries of disability-focused activities.

The alternative to the perpetuation of homogenising narratives about disability in the global South is the accounting of experiences and perspectives of PWDs that are grounded in the specific contexts in which they are expressed. Through these accounts, it is possible to understand disability in diverse and specific ways. One way to surface these experiences and perspectives is to focus on the practical concerns voiced by PWDs in specific contexts. Although contextually grounded research about meanings of disability has been produced in other areas of the global South (see Brégain 2016; Burck 1999; Chouinard 2014; Devlieger 1995), there has not yet been research of this kind in Western Zambia. Western Province is the traditional homeland of the Lozi people. Prior to the colonial interruption, the Lozi had developed an elaborate economy based on fishing, agriculture and cattle-herding on the rising and falling waters of the Zambezi River on the Barotse floodplain (Gluckman 1968). In modern Western Province, there are several ethnicities but Lozi language (Silozi) and culture dominate among the population of approximately 900 000 inhabitants (Central Statistical Office 2012). Approximately 85% of residents live in rural areas, while the remainder are spread throughout towns and the provincial capital of Mongu (CSO 2012). The main economic activities for people in Western Province – including city and town-dwellers – are subsistence fishing and farming supplemented by small trade. Within Zambia, Western Province had the highest consistent levels of poverty (80.4% – 83.3%) and extreme poverty (64.0% – 64.6%) in 2006 and 2010 as measured by the Zambian Central Statistical Office (2012). With geographic isolation, strong ties to traditional culture and high levels of poverty, there is reason to believe that Western Zambia has a unique context in which to experience disability. The purpose of this study therefore was to explore the expressed concerns of the members of two organisations of PWDs and their members in Western Zambia.

The study team comprised a PhD student and his advisory team. The PhD student and first author is a researcher and rehabilitation professional from Canada. The advisory team, senior academics, comprised three rehabilitation researchers working at Canadian universities, two with Canadian backgrounds and one originating from Brazil. The fourth member was an anthropologist based at a research institution in Zambia with a British background. The study activities were supported through the work of five paid research assistants, based in Zambia, who were post-secondary students from Western Province.

## Research method and design

### Study design

The study was guided by a qualitative constructionist methodology (Silverman 2006) with participatory (Herr & Anderson 2005) and critical considerations (Eakin et al. 1996) as part of a doctoral dissertation (Cleaver 2016). It is accepted in qualitative constructionist methodology that researchers and participants co-construct meanings (Silverman 2006). The key features of a critical social science perspective as described by Eakin et al. (1996) – reflexivity, assumptions and ideology, power, contradiction and dialectic – were an ongoing consideration throughout the study process, influencing the study framing, design, data collection and analysis. The sampling for this study was purposive to identify the organisation, but then also included the use of convenience sampling to deal with one unanticipatedly large organisation.

### Sampling and recruitment

The research fieldwork was conducted in Zambia’s Western Province between March and August 2014. The study team purposively recruited two organisations of PWDs (one urban and one rural) and their members to participate in this research. The decision was made to sample organisations rather than individuals alone to gain insight into disability organising and to allow for a more indigenous view of disability by relying on pre-existing structures. According to the preamble of the Zambian (2012) *Persons with Disabilities Act*, an organisation of PWDs exists to ‘promote and protect the interests of persons with disabilities’, while ‘most of its members are persons with disabilities’. Furthermore, it was foreseen that conducting the research with organisations would increase the likelihood that this research would complement ongoing initiatives.

To identify these organisations, contacts from local government offices shared connections with one organisation in a peri-urban neighbourhood of Mongu (urban) and another in a series of villages in an outlying district (rural). Contact for each of these organisations began with a request for a meeting with the leadership to discuss the organisation’s potential participation in the research. In both cases, the leaders agreed that participation was in the interest of the collective. We then approached members individually to discuss the research objectives and design and their individual participation. The original intent was to approach all the organisations’ members, but this strategy was revised for the rural organisation – to a combination of convenience and purposive sampling – when it became clear that the membership was larger than originally anticipated (Cleaver et al. 2017). Consistent to the structures of the organisations, parents participated on behalf of their children (18 years and under) with disabilities who were members of the organisations. Written consent was obtained from each participant during a meeting with a research assistant in a language of the participant’s choice. As a group, the research assistants were fluent in multiple Zambian languages that participants were likely to use locally on a regular basis, allowing the study team to allocate an assistant according to the participants’ language preferences. The key message at this initial meeting with the research assistant was that the purpose of the research was knowledge generation for practical purposes – such as informing the advocacy efforts of the organisations – and not the direct provision of resources to individual participants or the organisations.

### Participants

Two organisations and a total of 81 individual members participated in the study. Twenty-two of the participants were members of the urban organisation (see Table 1). This organisation identified its members according to a limited number of categories. For simplicity of presentation, the participants are described in Table 1 according to impairment types that approximate the organisation’s own categories. Eight of the members had family members participate in study activities with them or on their behalf.

**TABLE 1:** Participant demographics.

Variable	Urban group (*n* = 22)	Rural group (*n* = 59)
**Gender**	
Women and girls	11	24
Men and boys	11	35
**Age categories**	
18 years and under	6	9
19–64 years	11	14
65 years and over	5	24
Undeclared age	-	12
**Impairment type**†‡	
Physical	12	33
Visual	2	10
Intellectual	5	3
Hearing	3	2
Seizures	-	1
Communication	-	1

† , The urban organisation used its own system to categorise its membership; the members of the rural organisation described their disabilities in their own words, which we then used to create categories.

‡ , The sum total of impairment types in the rural organisation does not add up to 59: eight participants described themselves according to two impairment types of equal significance. Meanwhile, six participants described themselves as ‘elderly’ and 11 described themselves to be family members.

Fifty-nine participants were involved with the rural organisation (see Table 1). This group had a more fluid approach to membership, but had nine individuals (seven men and two women) who were consistently considered leaders. In addition, 50 ‘other members’ participated in the research. The rural group did not use a system to categorise the disabilities of the membership; instead, each member described the nature of his or her disability in his or her own terms. These descriptions varied widely, including references to body parts (e.g. leg, eyes), function (e.g. falling, I cannot see well, he does not work if he does not eat), disease states (e.g. leprosy, polio), healthcare interventions (e.g. they put wires in the legs), perceptions of deviance from expectations (e.g. not normal) and advanced age (e.g. elderly). We organised these individualised descriptions into impairment types in Table 1.

### Data collection

For each organisation, data were collected from an initial round of focus group discussions, followed by semi-structured individual interviews with many of the organisation members, and then a second round of focus group discussions (see Table 2 for the numbers of activities and individuals involved). The actual number of data collection activities varied according to circumstance and was negotiated together with the organisations at community meetings. Data collection activities were premised upon a gradual relationship-building process, structured and sequenced to include both collective and individual activities, and designed to minimise the extent to which the phenomenon of disability was defined by the researchers prior to entering the field. The first round of focus group discussions and the interviews were based upon questions about life with a disability, supported by probes about the positive and the negative aspects of life as it relates to disability. The second round of focus groups included discussion of specific activities, services and initiatives that were available in the communities where participants lived. The data collection activities were limited to the two participating organisations and did not include any other organisations.

**TABLE 2:** Data collection activities.

Variable	Urban group (*n* = 22)	Rural group (n = 59)
Round 1 focus group discussions (FGDs)	1 FGD – 18 participants†	4 FGDs:‡ A – 16 participants B – 12 participants C – 16 participants D – 15 participants
Interviews	20 individual interviews with 22 participants§	19 individual interviews¶
Round 2 FGDs	2 FGDs:†† A – 10 participants B – 8 participants	1 FGD – 7 participants‡‡

† , We conducted this FGD with a larger number of participants than we had originally planned. Disability leaders in Western Province had advised us that organisations of PWDs most typically have 10 members. With a membership of this size, we would be able to observe the organisation’s group dynamics through an activity that would be similar to their regular meetings and also the suggested size of an FGD (Maynard-Tucker 2000). Upon learning that the organisation was larger than we anticipated, we opted to prioritise the goal of replicating the regular meeting structure and held one large FGD.

‡ , According to the initial plans, all members of the purposively selected organisations should have been given the opportunity to participate. With its unexpectedly large membership, the rural group was subdivided according to four areas of residence for the first focus group discussion; each person participated in only one FGD, up to the capacity of the venue, while the nine leaders were spread among the four FGDs.

§ , In two cases, there were two family member participants who agreed to be interviewed together. For this reason, there were 20 interviews but 22 participants who completed an interview.

¶ , The 19 interviews were conducted with nine group leaders and 10 participants purposively selected based on their participation in the round 1 FGD.

†† , Because of crowding in the round 1 FGD, it was decided together with members during a community meeting that the round 2 would be more comfortable if the participants were divided into two groups.

‡‡ , The round 2 FGD in the rural group was only conducted with the leaders of the group. Seven of the nine leaders participated.

The first author led these activities speaking in English, while participants communicated in the language of their choice, and a research assistant performed real-time translation where necessary. Most participants opted to communicate primarily in the regionally dominant language of Silozi, although some participants chose English (verbal and written), Sign Language, or the less-common local languages Chimbunda and Makoma. All data collection activities were audio-recorded with the participants’ permission. Research assistant team members transcribed all speech in the actual languages spoken using a comprehensive transcription guide developed by the team.

### Data analysis

Data analysis began with a detailed review of all transcripts while listening to the audio files. The detailed review was used to guide an iterative analytic process, where initial ideas were used to generate subsequent questions of the data. Queries and answers generated during the iterative analytic process were transformed into visual schema and written documents to further organise ideas and eventually distil the themes presented in this article. Visual schema and written documents were used to generate further discussion and reflection as part of a process to gradually refine the analysis.

## Ethical considerations

This study was approved by the University of Toronto Health Sciences Research Ethics Board Protocol reference #: 29653), the University of Zambia Humanities and Social Sciences Research Ethics Committee, and the Zambian Ministry of Health. To reduce the burden of participation, participants’ travel costs were paid when there was motorised transportation available for hire, and a group meal was provided for large meetings and focus group discussions. Participating individuals and organisations are identified in this article in general terms to allow the reader to understand their situation without revealing the identity of the participants. In conducting the research, we followed critical (Eakin et al. 1996), postcolonial (Grech 2009; Meekosha 2011) and global health (Canadian Coalition for Global Health Research 2015) ethical research principles that consider the well-being and agency of participating communities with collective interests and concerns, and recognise that academic research is conducted within a dynamic of power relations.

## Results

The objective of this inquiry was to explore the expressed concerns of two organisations of PWDs and their members. The analysis of data collected during study activities showed that the accounts expressed by the participants were dominated by a single concern: poverty. Participants spoke about poverty in a particular way through the interconnected phenomena of *a need for material resources* and *a life of suffering*; this concern was inherently tied to the experience of disability.

### Material resources and suffering: A particular understanding of poverty

Participants in this research spoke frequently and emphatically about their suffering as part of a life with disability, and about their need for material resources. Effectively, these two ideas were interrelated, such that the lack of one was the cause of the other. As stated by an older woman with leprosy:

‘Since my disability has found me, there has been no one to build me a house, there is no one to find me food, there is nothing. Who will help me with this suffering?’

(Participant #R028, female, age described as ‘elderly’)

The reverse was also true: if a person received material resources, it would improve their situation and alleviate suffering. As stated by a man with a physical disability:

‘Like for me the way I feel, if I find someone giving me money, I will see that I have been helped and then they can improve my life.’ (U001, male, 32 years old)

**FIGURE 1:**
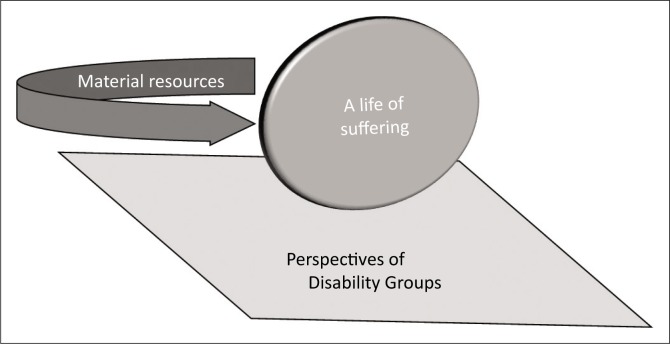
Poverty conceptualised as a two-sided coin.

The interrelationship between the two concepts of this particular understanding of poverty can be conceptualised as two sides of the same coin, where the sides are fused together, such that both are present even if only one is apparent at any given moment. In a similar fashion, the participants’ accounts of *a life of suffering* were attached to frequent references to *a lack of material resources* (see Figure 1). Conceptualising the participants’ concern as a single two-sided entity – poverty – facilitates the process of examining the relation of this concern to disability.

#### One side of the coin: Disability is a life of suffering

Participants overwhelmingly described the experience of their disability negatively, often using the Silozi terms *manyando* or *butata.* Collectively, these terms can be translated as problems, difficulties, a hard life or misery (Barotseland.net n.d.), but the research assistant interpreters most frequently translated these as *suffering*. For example, when organisation members participating in a focus group discussion were asked about their lives, an older man with leprosy stated ‘Kona kuli luikutwanga manyando’ which translated as: ‘We feel that we are really suffering’. In a different focus group discussion, another man with a congenital spinal deformity spoke about life for PWDs in his village as ‘a life of suffering’ (‘*bupilo bwa manyando*’).

Participants generally did not describe what was meant by *suffering*. When probed about the specifics of suffering in discussions about life with a disability, some participants identified aspects of anguish. For example, a woman with leprosy stated:

‘You cannot sleep all the night; you are there thinking about your suffering.’ (R005, female, 69 years old)

Another participant, a man with a visual impairment, stated:

‘Our disability, it is just suffering. We have a lot that is in the heart.’ (U002, male, elderly)

It seemed almost as if the meaning of *suffering* was sufficiently self-evident to participants to not require an explanation. Despite not describing the meaning of *suffering*, participants regularly related this to life with a disability. For example, the older man with leprosy declared:

‘It is disability that brings that suffering; when someone is not disabled, the person cannot suffer.’

#### The other side of the coin: A lack of material resources

The other prominent theme in the data was a *lack of material resources*. In this article, the term ‘material resources’ is a composite one, encompassing both (1) money and (2) items and services that can be purchased with money. In Table 3, there is a list of examples of material resources that participants mentioned in the context of discussing life with a disability. In many instances, the participants spoke about specific items (e.g. a new house, fertiliser, hair dryers), whereas in others there was discussion about money, and how items could be purchased if a person had access to money.

**TABLE 3:** Material resources that participants stated they did not have and/or needed.

Resource	Need identified
Tangible items	Food rations
	Hair dryers
	Sewing machines
	Fertiliser
	Fishing supplies
	Goods for resale
	Mill (the machine)
	Certificates of recognition
	Food and drink for visitors
	Housing
Services and expenses that could be covered if they were granted money	School expenses
	Tap costs
	Investment in a business
	Human resources to pay wages for: Firewood collection Cultivation Fishing assistance

Participants spoke about *material resources* as items that they needed but did not have, and were unable to acquire. During the data collection activities, participants regularly directed the discussion away from other topics introduced by the first author, directing the discussion towards material resources instead. Participants would sometimes advance this topic of discussion through straightforward declarations that a resource was needed or desired. In other instances, participants discussed not having something (or enough of something) and how this was a problem. In still other instances, participants spoke positively about situations where they had received resources in the past or could potentially receive them in the future.

One example of an account celebrating previous material resource distributions came from a man with leprosy. He described, in detail, the story of a foreign missionary who had formerly lived in his community, but had collected money in his country of origin to provide various support. As part of this account, he stated that:

‘In each place [*the missionary*] asked for one person who is educated who will be writing the report … and writing the names of people who were living in poverty. … Now he gave the order to say “These people now, you should build houses for them, these people who do not have one. A house and a kitchen and a fence”.’ (R002, male, 65 years old)

When discussing material resources – the lack thereof, the need for more or previous distributions – participants often related these to *suffering* or its alleviation. Just as participants had made connections between suffering and disability, they also connected suffering and material resources. As stated by a participant in a focus group discussion:

‘I wanted to talk on the problems people with disabilities face here. It is very hard, because here you cannot find money so that you survive.’ (R130, male, 32 years old)

In a different focus group discussion in the rural community, in response to the question ‘Is there anyone else who has something to add on what it is like to live with disability here?’, one man shouted the reply:

‘It is suffering because you cannot work so that you find food for you to eat.’ (R015, male, 50 years old)

Some material resources were presented as items the participants needed for their own personal use, such as better housing or food rations (i.e. bags of flour for the staple meal). As stated in a focus group discussion:

‘For me the only problem is my eyes. But I need someone to help me. There is no one to build a house, so what I want is just help from you. And you give me food.’ (R127, male, elderly)

In other cases, the reference to the resource was with respect to the utility of material resources for earning more income. When asked about the positives of having a disability in a focus group discussion, a woman with a physical disability that limited her capacity to walk said:

‘I am even talented on hair styling. I am a hair stylist. I can plait any type of a style for hair any type that a person wants. I can do it, but I do not have money to start my own salon.’ (U010, female, 26 years old)

For this woman, having a salon meant renting a market stall and purchasing some hand-held hair dryers – modest investments, but ones that required more money than she could access.

Occasionally, participants were ambiguous as to whether the resources would be used for personal use or as income generation. Examples included needing money to pay an individual to collect firewood, to cultivate a garden or to assist with fishing. This is exemplified by a participant with a visual impairment, stating:

‘[*Before I contracted cerebral malaria*] I was able to cultivate or plough with my own hands but these days I cannot see. I was able to go fishing, paddling in the canals, but now I cannot. I can do these things if I have someone [*to help me do them*]. And that person needs to be paid. But where am I going to get the money?’ (U007, male, 67 years old)

### Ways in which the poverty coin linked to disability

Participants presented the concern of poverty as inherent in their experience of disability. During initial interactions, participants generally identified their disability in very similar terms to the specific embodied state of having an impairment (World Health Organisation 2002). They then linked this state to *the poverty coin* in patterned ways. Two of these patterned ways were (1) impairments impeding resource acquisition, resulting in suffering, and (2) poverty as an integral part of the experience of disability.

#### Impairments impede material resource acquisition, resulting in suffering

In some of the participants’ accounts, the link between impairment-related functional limitations, a lack of material resources and a life of suffering was described as a series of causal steps. Often, the situation involved only one step, where an individual’s impairment reduced employment opportunities or the ability to farm for food or income. As stated by a man with difficulties walking and speaking:

‘We are really living in poverty. Like the others have said, when there is work, we people who are disabled cannot do it. Even when you try to go there, they will tell you, “You, you cannot do it.” And that is really the truth, that you really cannot.’ (R015, male, 50 years old)

Another participant spoke about how his inability to walk limited the activities that he could do and the compensation he could earn from these activities when clients chose to not pay:

‘Like for me I cannot say “I can go and cultivate, I can go and fetch water,” or “I can go and do this.” Because even this thing in which I am seated, I need to find someone push it. But God has given me the knowledge of how to repair shoes. But again, if a request a certain price to say, “maybe I can manage to buy a cup of flour or something, so that I feed myself,” they refuse also. Now them, they want to be able to choose how much to give me. Because they know that I have no option, there is no way I can refuse their money, and that “I can give him this money” because they know I am disabled I have no option, I just have to accept the amount.’ (R009, male, 59 years old)

In some cases, it was the impairments of children who kept the parents at home. These limited opportunities to acquire or generate resources are thus the primary and direct explanations that underlie the participants’ lives of suffering. The mother of an 11-year-old girl considered to have an intellectual disability by the organisation gave an example of this when she expressed (U009):

‘I cannot do a business which will need me to walk or travel to go very far, leaving her. I cannot leave her.’

Participants spoke of how an infusion of material resources could offer an alternative path to success without requiring any change to their own specific impairment, or the impairment of their family member. For example, a gift of a sewing machine would allow income generation in the home so that the mother quoted above could simultaneously care for her child with a disability while earning money to eliminate suffering, thereby overcoming the problems of poverty.

Another example of this phenomenon came from a woman with a physical disability limiting the use of her hands. This woman spoke about how she had previously purchased fish and then travelled with it elsewhere in the country to re-sell it at a profit. As she had used the money to pay for school fees for a child, she no longer had the money to generate income. Nonetheless, the participant was confident that her fortunes would change with monetary support to travel to the flood plains for the purpose of buying fish:

‘Right now I do not have the money; if I did I would have gone even a long time ago.’ (U006, female, 39 years old)

In other cases, there were multiple steps separating the participants’ current situation of poverty from their aspirations. An example of this was the one woman with a physical disability who was hoping to find money to buy hair dryers and open a salon. According to the woman, her difficulties walking reduced her income generating capacity, which, in turn, inhibited her from being able to amass capital to start a business which would provide further income to pay for a return to school to study to be a professional. The woman’s account of her current situation was one of stagnation and struggle, working as a hairdresser for salon owners for little money to provide basic needs for her son. By contrast, a positive cycle could have been activated through loans or grants of money or materials that would have allowed her to start the business.

#### The poverty coin as an integral part of the experience of disability

In many accounts, the relationship between a lack of material resources and suffering was not a series of connected steps, but one where disability was integrally understood to apply to individuals who identified as having an impairment while living the two-sided coin of poverty. When invoking this pattern, participants spoke about poverty and disability interchangeably, as if they were using two synonymous words to describe a single phenomenon.

One example of this phenomenon occurred during a focus group discussion. The participants were speaking about the suffering experienced by PWDs. One man with a physical disability added:

‘What I have seen in life is if you are disabled but you are working, you are doing a business, people will respect you for that. For you who does not have anything and you are disabled, you are poor. No one can respect you, even the family members cannot respect.’ (U003, male, 68 years old)

From the quote above, it might seem that disability and poverty could be teased apart such that a person with a disability *doing a business* would garner respect. The first author asked a follow-up question to confirm that this was the case:

*First author:* I would like to find out how things are different for persons with disabilities who own businesses as compared to people without disabilities who own businesses?

*Participant:* We differ because some people, maybe when they come to visit you if you are doing a business you will be able to give them something but if you do not have a business which you are running, even when they ask you something you cannot give them anything because you do not have, and so you are not regarded. … All [of the problems of poverty are] brought because of being disabled.

Instead of following the first author’s question of comparing the experience of business ownership and wealth among persons with and without disabilities, the participant responded with a different comparison: the poor and disabled as compared to the rich and non-disabled. Although it seemed possible to isolate comparisons of wealth (regardless of disability) or disability (regardless of wealth), additional discussion demonstrated that these notions were expressed and understood in nearly interchangeable terms.

In the account of another participant with a physical disability, wealth and function were also bundled and contrasted to poverty and dysfunction. Following the participant’s self-identification as *a lame person [sic]*, the first author asked, ‘If starting tomorrow everyone forgot that you are “a lame person,” but nothing else changed, would your life be the same or would it improve?’ The man’s reply demonstrated how perceptions of *lameness* were simultaneously premised on a poverty of possessions as well as an inability to do things:

‘Ah, on that way I was going to be grateful because maybe this mocking is coming because of what I have or what I do not have because of being poor. But if they see me that, ‘that person this time has got this; he is able to do this,’ they will give me respect.’ (U005, male, 41 years old)

Given the man’s situation of impairment-poverty, he considered the organisation of PWDs to provide him with a ‘very big help’. This caused us to wonder about the strategies used by this man’s neighbours, who from casual observation seemed to be equally poor, but likely did not claim to be disabled. From the perspective of this man, the situation of persons without disabilities was not one that interested him:

*First author:* Your neighbours who do not have disabilities, do they form groups or other things to try to make their voices stronger?

*Participant:* I cannot answer that question because I do not know how they are living.

As is seen from these examples, it was as if it was only PWDs who could be poor. Meanwhile, as demonstrated with the data above, being rich (i.e. doing a business, having things) was the categorical opposite of being disabled. In parallel, the possibility of poverty among people without disabilities was not a phenomenon of interest for the participants. When participants spoke about poverty as being integrally part of disability, it seemed taken for granted that the coexistence of needs and the inability to meet them were the defining elements of being a person with a disability. The proposed solutions were therefore gifts of material resources to directly meet their needs.

## Discussion

This study is the first to explore disability through a contextually grounded investigation in Western Zambia. We asked PWDs in this context about their lives with disabilities (including positive and the negative aspects thereof) and the specific activities, services and initiatives that were available in the communities where they lived. In response, a wide variety of participants from both organisations referred to living a life of suffering and of having a lack of material resources. Taken together, these references can be conceptualised as poverty. The frequency and emphasis of the participants’ concerns, and the ways in which these were linked to disability, demonstrate the centrality of poverty in the collective meaning of disability in this context. This study offers a rare perspective of disability in Western Zambia, but also has important implications for the literature on disability theory and the relation of disability and poverty, in addition to implications for policy and practice.

### Implications for literature on disability theory

The participants’ concern for poverty could have been consistent with any one of multiple models of disability, particularly the charity and medical models (Clare 2001). Consistent with the charity model, some participants spoke about poverty and disability as being conceptually intertwined, as if PWDs could never expect to work their way out of poverty. Consistent with the medical model, some participants spoke about impairments as being a root cause of their poverty.

In comparing the findings of this study to literature about disability in the global South, it is remarkable that participants’ concerns *were not* aligned to the pervasive myths (Ingstad 1999) that we identify collectively as ‘the traditional model’. Participants did not report anecdotes of PWDs being hidden away by family members, nor did they discuss the infanticide of children with disabilities. The design of this study does not allow us to conclude that hiding and neglect are completely absent in Western Zambia; it is possible that these practices occur, yet they were not front-of-mind in the collective consciousness of those most likely to be concerned by them. It is also remarkable that participants in this study expressed numerous and emphatic perspectives about the effects of their disabilities, but very little about the root causes. During the interviews, we asked participants about the history of their disability, including the way it began. The participants answered our questions, but they devoted little interest and consideration to explaining their views on the causes of their disabilities. The implication of this study for literature on disability theory is to provide yet another contextually grounded example to contradict the homogenising narratives of influential ‘international’ actors, particularly the narrative that most people in the global South consider disability to be caused by supernatural phenomena (Grech 2015; Miles 2007).

### Implications for literature on disability and poverty

This study was designed to be exploratory such that the concerns of the participants could emerge, regardless of what those might be; while we anticipated at the outset of the study that concerns aligned with the ‘traditional model’ might be prominent, we did not foresee the centrality of poverty. Through data analysis, it became clear that these participants spoke about poverty in various ways. These patterns provide insight about how the participants might understand poverty and its relationship to disability. Issues of disability and poverty have garnered increased attention in recent years through specific attention in the United Nations (2006) Convention on the Rights of Persons with Disabilities (UNCRPD) and through research and publications (e.g. Eide & Ingstad 2011; Pinilla-Roncancio 2015). Despite not being designed to focus on poverty, this study adds perspectives to the literature on the topic.

#### Building a qualitative understanding of poverty from participants’ expressed concerns

Much research on disability and poverty has taken a turn away from an economic resources approach (Palmer 2011), adopting instead a capabilities approach (Muderedzi et al. 2017; Sen 1993; Trani et al. 2017). In this study, the expressed concerns of the participants were clearly focused on material resources, a finding that is consistent with the economic resources approach.

As it was not a focus of this study to quantify indicators of poverty, we did not attempt to accurately compare the individual or household situations of research participants with each other or persons without disabilities. However, from our informal observations in the community, it appeared that most community members (with and without disabilities) were of similarly poor economic status. Indeed, in a Zambia-wide study that did compare individuals and households with and without disabilities, the overall findings were that household wealth was similar, although there were important differences with respect to education and employment levels, with poorer access for PWDs (Eide & Loeb 2006; Trani & Loeb 2012). If the results of the Zambia-wide study are consistent with the two communities where the participants lived, the expressed concerns presented here could be indicative of widespread poverty that is understood through economic resources but felt more deeply by PWDs because of multidimensional considerations (Trani & Loeb 2012).

#### The relationship of disability and poverty

**FIGURE 2:**
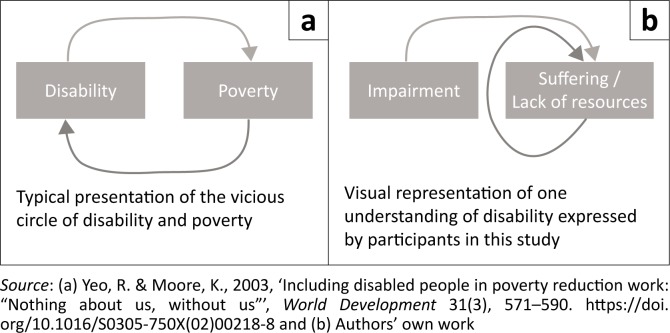
Typical and alternative presentations of the relationship of disability and poverty.

Most commonly, disability and poverty have been presented as a *vicious circle* (Yeo & Moore 2003), such that experiencing one increases the probability of experiencing the other. If we understand disability to be impairment-related function, many participants in this study framed their situation in a fashion that is similar to one half of the vicious circle: that their disability contributed to their lack of material resources, which was the foundation of their lives of suffering (see Figure 2). When speaking about things in a cyclical manner, participants did not draw upon a disability-causes-poverty-causes-disability framework but instead spoke about how a lack of material resources made it difficult to generate or acquire additional material resources; in effect, that poverty-begets-poverty (Pagani 2007).

It must be noted that many of the participants did not articulate the narrative that their impairment-related function initiated their situation of poverty; instead, impairments were part of the description of disability, but only coincidentally part of the experience. Meanwhile, poverty was *integral* to the experience of disability. As shown in the alternative presentation of disability and poverty (see Figure 2), when disability is understood in this manner, there seems to be only minimal, if any, ongoing connection between disability and impairment.

Furthermore, a *poverty as integral to disability* perspective can be related to other descriptions of the relation of disability and poverty. Two years after writing about the vicious circle, Yeo (2005:34) proposed that disability and poverty ‘would be better described as interlocking circles’, because of the common experiences of marginalisation, isolation, deprivation and lack of access. Yeo’s (2005) proposal might be more similar to the narratives of participants in this study, except that interlocking circles imply that there can be poverty-without-disability and disability-without-poverty. Instead, participants spoke of a disability or poverty in which everyone who was disabled was poor and where poverty was experienced uniquely by the disabled. In empirical research in a rural area of South Africa, Hansen and Sait (2011) proposed that research participants experiencing extensive exclusion and *social suffering* identified themselves as disabled. Hansen and Sait’s (2011) proposal would seem to fit the data from this study well.

### Implications for policy and practice

In the results of this study, poverty was more prominent in the accounts than impairment and function, and also more prominent than other possible considerations. The implication for policy and practice is to mainstream the concern of poverty into disability activities.

One specific example of the way that poverty could be mainstreamed into disability activities is to compare these perspectives to a prominent template for programming for PWDs: The community-based rehabilitation (CBR) matrix (Khasnabis & Motsch 2010). Of the five components of the CBR matrix, the participants’ concern was weighted very heavily towards the livelihood component. Furthermore, participants spoke about each of the elements of the livelihood component with regular reference to self-employment, waged employment, financial services and social protection.

Meanwhile, the participants’ references to other components of the CBR matrix were generally subservient to livelihood considerations. An example of this is health. Concerns related to the health component of the CBR matrix were rarely brought forward by participants. When participants were asked questions related to the health component elements of disease prevention, medical care and rehabilitation, the participants responded with general disinterest, even when acknowledging that these services were not available to them.

## Considerations

We conducted this study in a particular context. In qualitative constructionist research, context is not seen as a source of bias that interferes with the accuracy of research results and must therefore be eliminated (Silverman 2006). Instead, research is seen to be constructed in contexts and it is the role of the researcher to consider the context as part of the interpretation. The larger context of this study was Western Zambia, a jurisdiction that we describe in detail in the *Research method and design* section of this article. Within the larger context, the research was produced in a more particular context: through interaction between a researcher with a specific appearance and life history and members of local organisations of PWDs.

In some ways, this particular context constrained possibilities for the project. Examples of possibilities constrained by researcher–participant positionality in this project included the difficulties of fulfilling principles of authentic partnership and shared benefits (‘author’ et al. 2016), both of which are important participatory research considerations (Herr & Anderson 2005). In contrast, the respective positionality of the researcher could have been productive, increasing the interest of organisation members to participate in the research and stimulating them to share their experiences – albeit in certain ways. In this particular context, it would have been reasonable for the participants to have seen the first author as a person who might be connected to resources. Accordingly, it would have been relevant and practical for the participants to emphasise their concern for poverty, a concern that the first author could be seen to be well positioned to address.

The study’s particular context does not mean that the participants’ concern for poverty was an artefact produced only in the presence of the first author: the consistent, persistent and detailed accounts of disability experienced as suffering and material resource deprivation made for a very compelling concern. Moreover, there is evidence to suggest that the two organisations – each of which had a history pre-dating the arrival of the first author – evolved according to the availability of outside programming, especially programming related to material resource acquisition (Cleaver et al. 2017). Given the North-South dynamics of the contemporary postcolonial world, the particular context of this research might not be all that particular; indeed, it could merely be one case among many where the concept of disability is produced and reproduced interactively through power and resource imbalances.

Also, this was a context where the research participation was limited to those who were members of organisations. The focus on collectives meant that it was not possible to explore the first-hand experiences of PWDs in Western Province who were not attached to an organisation, which would, in turn, exclude the participation of any PWDs who were being socially isolated by family members at the time. Recognising that ‘international actors’ engaged in disability activities in the global South have come to expect accounts of stigma, shame and the isolation of PWDs, we are compelled to declare that this study was not designed to seek out such cases, if they exist.

A final consideration of the particular context of this study is the analytic impact of approaching organisations as participants. In part, it was because of the collective orientation that the analysis focused initially on the commonalities within and between the organisations, rather than searching for contrasts between the collectives or individual members. The analysis of these data has not included potentially important differences within the groups, such as that of gender, age, wealth, social status or ‘disability type’. Considering that the experiences of PWDs are not homogenous (Miles 2003), various sub-group and individual analyses could yield important insights.

## Conclusion

The members of two organisations of PWDs in Western Zambia who participated in this constructionist research expressed overwhelming concern with poverty. This concern was expressed through the interrelated phenomena of a life of suffering and a lack of material resources (i.e. money or things that could be exchanged for money). These findings contribute to the growing interest in poverty and disability through the articulation of contextually grounded formulations of the ways in which these phenomena are conceived and the identification of associated policy and practice implications. Finally, it was notable that the concerns of these PWDs in Western Zambia were incongruent with the notion that understandings of disability in the global South are dominated by ‘the traditional model of disability’.

## Footnotes

1 To some, the conceptualisation of a global South inappropriately homogenises much of the world and reifies the dominance of high-income countries. We recognise this critique. Nonetheless, we find analytic value in considering how the legacy of colonialism has had similar effects in different places. Following the work of others writing in the field of postcolonial disability studies (e.g. Shaun Grech and Karen Soldatic), we see the commonalities of this legacy in multiple locations in the global South.
